# Detection of gastric cancer-associated microRNAs on microRNA microarray comparing pre- and post-operative plasma

**DOI:** 10.1038/bjc.2011.588

**Published:** 2012-01-19

**Authors:** H Konishi, D Ichikawa, S Komatsu, A Shiozaki, M Tsujiura, H Takeshita, R Morimura, H Nagata, T Arita, T Kawaguchi, S Hirashima, H Fujiwara, K Okamoto, E Otsuji

**Affiliations:** 1 Division of Digestive Surgery, Department of Surgery, Kyoto Prefectural University of Medicine, 465 Kajii-cho, Kawaramachihirokoji, Kamigyo-ku, Kyoto 602-8566, Japan

**Keywords:** microRNA, plasma, gastric cancer

## Abstract

**Background:**

Recently, it was reported that plasma microRNAs (miRNAs) are low-invasive useful biomarkers for cancer. We attempted to isolate gastric cancer (GC)-associated miRNAs comparing pre- and post-operative paired plasma, thereby excluding the possible effects of individual variability.

**Methods:**

This study was divided into four steps: (1) microarray analysis comparing pre- and post-operative plasma; (2) validation of candidate miRNAs by quantitative RT–PCR; (3) validation study of selected miRNAs using paired plasma; and (4) comparison of the levels of selected miRNAs in plasma between healthy controls and patients.

**Results:**

From the results of microarray analysis, nine candidate miRNAs the levels of which were markedly decreased in post-operative plasma were selected for further studies. After confirmation of their post-operative marked reduction, two candidate miRNAs, miR-451 and miR-486, were selected as plasma biomarkers, considering the abundance in plasma, and marked decrease in post-operative samples. In validation, the two miRNAs were found to decrease in post-operative plasma in 90 and 93% of patients (both *P*<0.01). In comparison with healthy controls, the levels of both miRNAs were found to be significantly higher in patients, and the area under the curve values were high at 0.96 and 0.92.

**Conclusion:**

Plasma miR-451 and miR-486 could be useful blood-based biomarkers for screening GC.

Gastric cancer (GC) is the second leading cancer-related cause of death globally (Jemal *et al*, 2011). Recent advances in diagnostic techniques and peri-operative management have increased the early detection of GC and decreased the mortality rate. However, patients with advanced disease still frequently develop recurrent disease after extended radical resections, and consequently demonstrate extremely poor survival rates (Hohenberger and Gretschel, 2003; Hartgrink *et al*, 2009). Thus, the primary tumours must be detected at an early stage, and recurrent disease must be diagnosed when it is still minimal or clinically occult, in order to improve the cure rates for patients with GCs, like breast or lung cancer (Mascaux *et al*, 2010).

Recently, several studies have demonstrated that microRNAs (miRNAs), which are involved in tumourigenesis and the development of various cancers (Lu *et al*, 2005; Calin and Croce, 2006; Lee and Dutta, 2009), are stably detectable in plasma/serum (Chen *et al*, 2008; Mitchell *et al*, 2008). Accumulating reports demonstrated that circulating miRNAs could be good candidates for non-invasive diagnostic biomarkers in various cancers (Chan and Lo, 2007; Mitchell *et al*, 2008; Zen and Zhang, 2010; Kosaka *et al*, 2010a). As for GCs, we have already reported that detection of circulating miRNAs might provide new complementary tumour markers for cancer screening (Tsujiura *et al*, 2010). Some studies, however, have shown that miRNAs regulate specific genes broadly involved in a wide range of physiologic pathways (Ambros, 2004; Bartel, 2004), such as development, cell death, cell proliferation and metabolism, which suggests the possible effects of individual variability and also age-related biological change on plasma miRNA expression study (Hackl *et al*, 2010; Noren *et al*, 2010).

These findings prompted us to search for cancer-associated plasma miRNAs in an analytical approach that is unaffected by individual variability of miRNA expression. In this study, we isolated GC-associated miRNAs in plasma on a miRNA microarray by comparing pre- and post-operative paired plasma samples, and confirmed the levels of the isolated miRNAs in pre- and post-operative paired plasma samples in a large cohort. We also compared the plasma miRNA levels of patients with those of healthy controls to assess the diagnostic value of these biomarkers in patients with GCs.

We suggest that the miRNAs detected based on these concepts are valuable biomarkers for the effective detection of recurrence or early cancer, because the change of these miRNAs will be affected by the reduction of cancer.

## Patients and methods

### Patients and samples

A total of 56 plasma samples were collected from the GC patients, who underwent gastrectomy between January 2009 and August 2010 at Kyoto Prefectural University of Medicine. The collections were performed at least two times, before and 1–2 months after the operation. Thirty control samples were collected from healthy volunteers with no cancerous diseases. Relevant clinical and survival data were available for all patients. Written informed consent was obtained from all patients after approval by the local ethics committee. In some patients whose plasma miRNA levels were determined, formalin-fixed paraffin-embedded tumourous and non-tumourous tissue samples were also examined. The macroscopic and microscopic classifications of tumours were based on the UICC/TMN staging system (Sobin *et al*, 2010).

### Stock of plasma samples

Immediately after collection in sodium heparin tubes (BD Vacutainer, Franklin Lakes, NJ, USA), the blood samples were subjected to isolation of cell-free nucleic acids using a three-spin protocol (1500 r.p.m. for 30 min, 3000 r.p.m. for 5 min and 4500 r.p.m. for 5 min) to prevent contamination by cellular nucleic acids. The plasma samples were stored at −80 °C until further processing.

### RNA extraction

Total RNA of plasma sample was extracted from 400 *μ*l of stocked plasma using a miRVana PARIS Kit (Ambion, Austin, TX, USA), and eluted into 100 *μ*l of pre-heated (95 °C) Elution Solution according to the manufacturer’s instructions. The tissue sample was obtained from tumour tissue or adjacent, histologically non-tumourous tissue. Total RNA of these tissues was extracted from four slices of 15-*μ*m-thick formalin-fixed paraffin-embedded tissues (total thickness of 60 *μ*m) using a Recover All Total Nucleic Acid Isolation Kit (Ambion), and finally eluted into 60 *μ*l of Elution Solution according to the manufacturer’s instructions. The RNA samples were stored at −80 °C until further processing.

### Study design to develop novel plasma miRNA biomarkers

**Figure 1:**
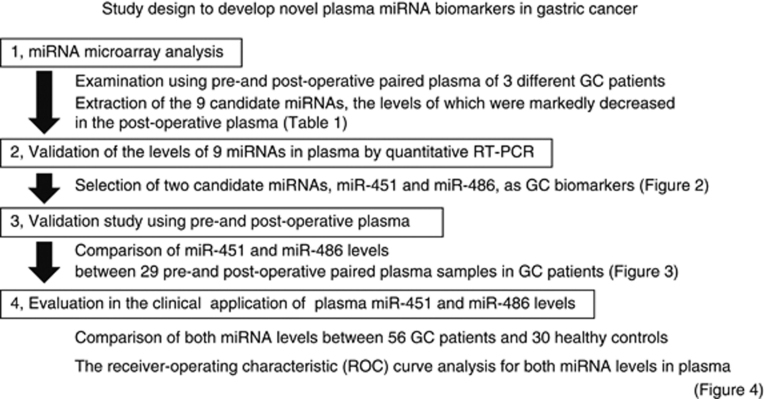
Study design to develop novel plasma miRNA biomarkers. This study was divided into four steps.

The study design is summarised in Figure 1. This study was divided into four steps: (1) microarray analysis comparing pre- and post-operative plasma miRNAs in 3 different GC patients; (2) validation study of candidate miRNAs by quantitative RT–PCR; (3) validation study of selected miRNAs using pre- and post-operative paired plasma in 29 GC patients; and (4) comparison of the levels of selected miRNAs in plasma between the 30 healthy controls and 56 GC patients.

### miRNA microarray analysis

The pre- and post-operative plasma samples of three different GC patients were selected for the microarray analysis. Clinicopathological features are summarised in Supplementary Table S1. All of the three GC patients underwent macroscopic radical operation, and had not developed recurrence by August 2011. Microarray analyses were performed using the 3D-Gene miRNA microarray platform (TORAY, Kamakura, Japan) in plasma samples (Nagino *et al*, 2006; Hisaoka *et al*, 2011). RNA extraction was performed according to the manufacturer’s instructions. The amount of total RNA in plasma was too small, and so 2 of 4 *μ*l of extracted total RNA from 300 *μ*l of plasma samples were used in the microarray experiments. This RNA was labelled with Hy5, and hybridised at 32 °C for 16 h on the 3D-Gene chip. The 3D-Gene miRNA microarray can mount >1000 miRNAs based on the Human miRNA Version15 of MirBase (http://microrna.sanger.ac.uk/). We analysed the data of microarray analysis, and selected nine candidate miRNAs whose levels were significantly downregulated in post-operative plasma samples compared with those pre-operatively for further detailed analysis.

### Protocols for the detection of miRNAs

Reverse transcription reaction was carried out with a TaqMan MicroRNA Reverse Transcription Kit (Applied Biosystems, Foster City, CA, USA), and the levels of miRNAs were quantified in duplicate by qRT–PCR using human TaqMan MicroRNA Assay Kit (Applied Biosystems) in accordance with previously described protocols (Komatsu *et al*, 2011). In brief, quantitative PCR was run on a 7300 Real-time PCR system (Applied Biosystems) and the cycle threshold (*C*_t_) values were calculated with SDS 1.4 software (Applied Biosystems). The levels of miRNAs in plasma were calculated using the concentration (amol *μ*l^–1^) on a standard curve constructed with the use of synthetic miRNAs, the miRVana miRNA Reference Panel (Ambion), because the stable and suitable PCR controls of plasma sample had not been reported (Reid *et al*, 2011). The expression of miRNAs from tissue samples was normalised using the ΔCT method relative to U6 small nuclear RNA (RNU6B). The change in gene expression was calculated with the equation 
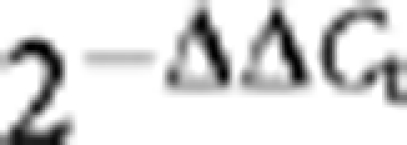
 (Livak and Schmittgen, 2001; Pfaffl, 2001).

### Statistical analysis

The Wilcoxon test was used to compare the paired plasma samples obtained before and after operation, and the Mann–Whitney test was used to compare the difference in the plasma miRNA concentration between the cancer group and the healthy control group. *P*-value <0.05 was considered significant. Receiver-operating characteristic (ROC) curves and the area under the ROC curve (AUC) were used to assess the feasibility of using plasma miRNA as a diagnostic tool for the detection of GC. Youden index was used to determine the cut-off value for the plasma miRNA concentrations (Akobeng, 2007).

## Results

### Results of miRNA microarray analysis and validation study on quantitative RT–PCR

The list of miRNAs the levels of which were markedly decreased in post-operative plasma samples compared with those pre-operatively are shown in Table 1. Some miRNAs such as miR-25, miR-17, miR-106a, miR-92a and miR-20a have been reported to be upregulated in GC tissues; however, others such as miR-451 and miR-486 have been reported to be downregulated in GC tissues. Some other miRNAs have not been previously reported to be associated with GC tissues. We selected nine miRNAs for validation study of microarray results and further detailed analyses as novel candidate biomarkers (Table 1, arrowheads).

**Figure 2:**
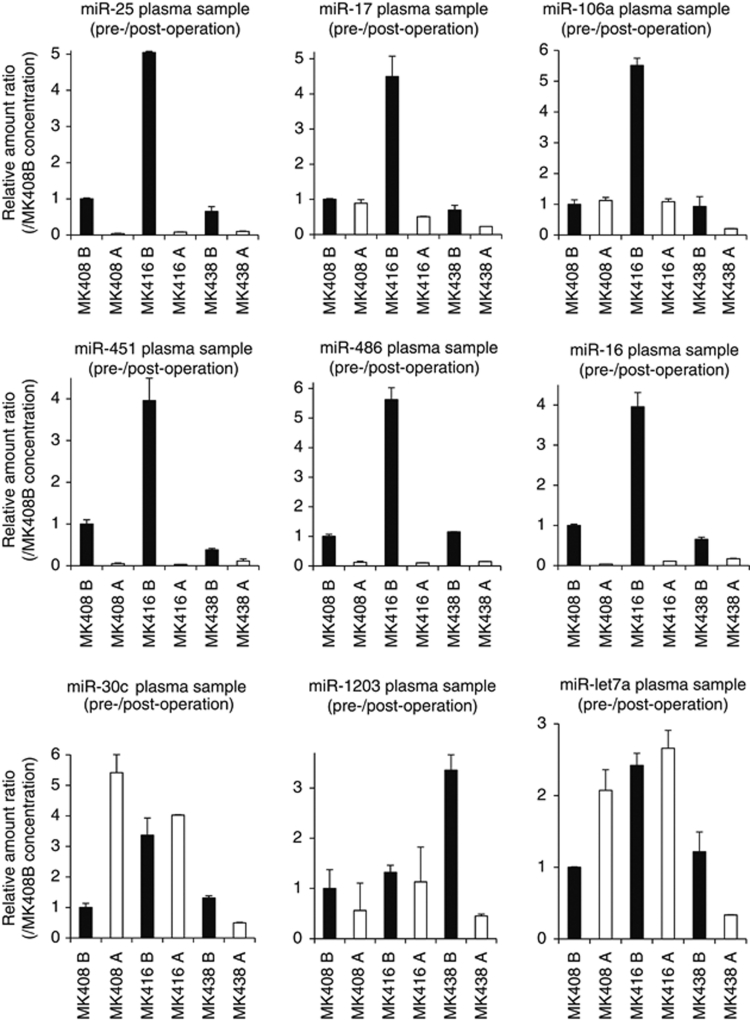
Comparison of the levels of miRNA in pre- and post-operative paired plasma samples in qRT–PCR. Quantitative RT–PCR assay was performed to validate the microarray data of selected miRNAs. The paired (pre- and post-operative) plasma samples of three GC patients were the same sets examined in the miRNA microarray analysis. The results of qRT–PCR showed the same tendency as those of microarray analysis for most miRNAs (7 of 9 miRNAs). Results are shown with means±s.d. (bars) relative to the level for pre-operative plasma sample, MK408B. Each column shows the mean for duplicate experiments. A, post-operative sample; B, pre-operative sample.

The results of microarray analysis were validated using the same paired (pre- and post-operative) plasma samples for these nine miRNAs by quantitative real-time RT–PCR (qRT–PCR) (Figure 2). The validation study demonstrated that the results of qRT–PCR showed the same tendency as those of microarray analysis for most miRNAs (7 of 9 miRNAs). Then, two candidate miRNAs, miR-451 and miR-486, were selected as novel plasma biomarkers for GC patients, considering their abundance in plasma samples and marked decrease in post-operative samples.

### Validation study of pre- and post-operative paired plasma samples

**Figure 3:**
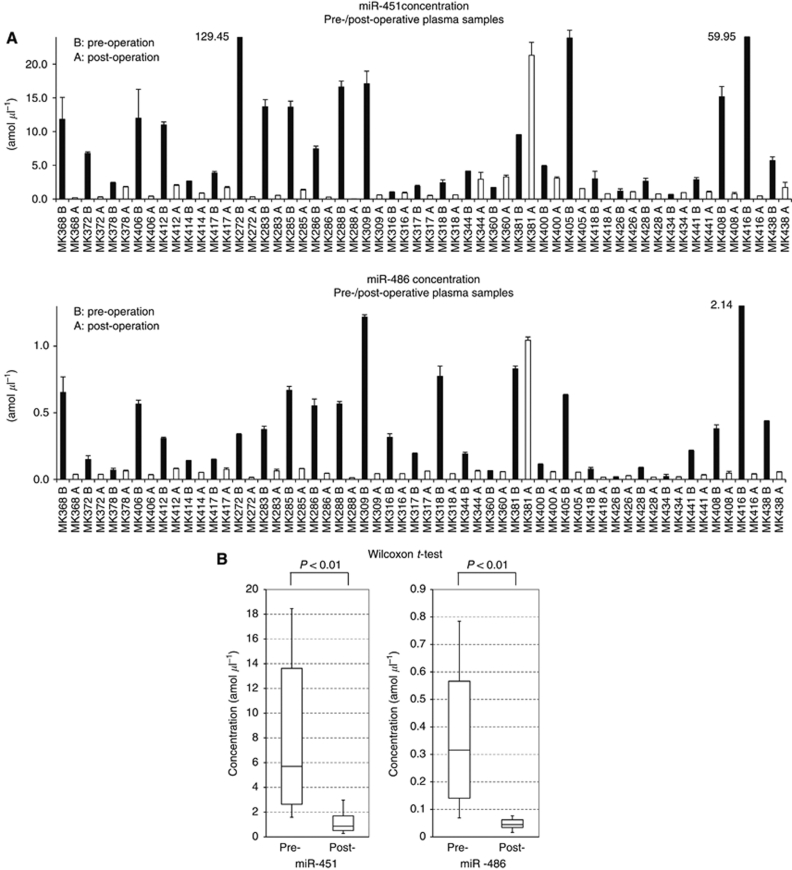
Results of plasma miR-451 and miR-486 in pre- and post-operative paired samples. The concentrations (amol *μ*l^–1^) of miR-451 and miR-486 in pre- and post- operative plasma samples of 29 GC patients were examined by qRT–PCR. (**A**) The concentrations of miR-451 and miR-486 in post-operative plasma were lower than those pre-operatively in 90% and 93% of GC patients, respectively. Each column shows the mean for duplicate experiments; bars, s.d. Marked high data are indicated by numerals and columns are abbreviated. A, white-columns represent post-operative samples; B, black-columns represent pre-operative samples. (**B**) Box plots of the pre- and post-operative plasma miRNA concentrations (both *P*<0.01, Wilcoxon *t*-test). The upper and lower limits of the boxes and the lines inside the boxes indicate the 75th and 25th percentiles and the median, respectively. The upper and lower horizontal bars denote the 90th and 10th percentiles, respectively.

**Figure 4:**
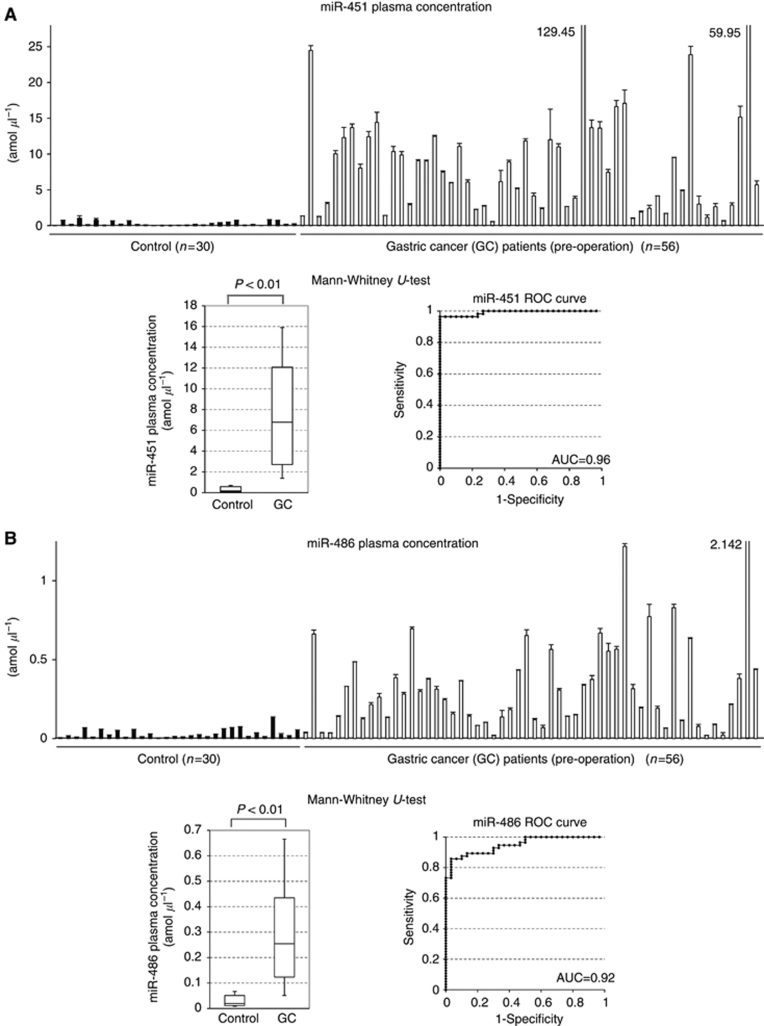
Comparison of plasma miR-451 (**A**) and miR-486 (**B**) between 30 healthy controls and 56 GC patients. The results of comparison between healthy controls and GC patients are presented for plasma miR-451 (**A**) and miR-486 (**B**) concentrations by qRT–PCR. Each column shows the mean for duplicate experiments; bars, s.d. Marked high data are indicated by numerals and columns are abbreviated. Box plots show that the plasma concentrations of both miRNAs were significantly higher in GC patients than in healthy controls (both *P*<0.01, Mann–Whitney *U*-test). Receiver-operating characteristic curve analyses on the concentrations of both miRNAs are also shown. The AUC values were 0.96 for miR-451 and 0.92 for miR-486.

Next, the levels of miR-451 and miR-486 were examined at a large scale for pre- and post-operative paired plasma samples in GC patients by qRT–PCR. The levels of both miRNAs are shown in absolute amounts per microliter (Figures 3A, 4A and, B). The concentration of miR-451 in post-operative plasma was decreased in 26 cases (26 out of 29; 90%) compared with that in the pre-operative sample, and miR-486 in 27 cases (27 out of 29; 93%) (Figure 3A). In addition, the concentrations of miR-451 and miR-486 were significantly decreased in post-operative plasma compared with those pre-operatively (both *P*<0.01, Wilcoxon *t*-test, Figure 3B).

### Comparison of the levels of miR-451 and miR-486 in plasma between the healthy controls and GC patients

Finally, the levels of miR-451 and miR-486 were compared in plasma samples between 30 healthy controls and 56 GC patients including the 29 pre-operative cases in the paired sample analysis described above. Clinicopathological features of these GC patients and the concentrations of pre-operative plasma miR-451 and miR-486 are shown in Supplementary Table S2.

The levels of both miRNAs were found to be significantly higher in GC patients than in the healthy controls (both *P*<0.01, Mann–Whitney *U*-test, Figures 4A and B). The ROC curve analyses on the concentrations of plasma miR-451 and miR-486 showed that the AUC were high (0.96 and 0.92, respectively) and thus useful for detecting GC patients. In these models, optimal cut-off points were indicated at 0.97 amol *μ*l^–1^ (sensitivity 96% and specificity 100%) and 0.073 amol *μ*l^–1^ (sensitivity 86% and specificity 97%) for miR-451 and miR-486, respectively (Youden index). These results suggested that these two miRNAs could be candidate plasma biomarkers for the screening and/or evaluation of treatment of GC patients.

### Comparison of miR-451 and miR-486 expressions between non-tumourous and GC tissues

Contrary to our expectation, the miR-451 expression was found to be significantly lower in GC tissues than in surrounding normal tissues (*P*=0.012), and the miR-486 expression was also found to be relatively lower in GC tissues than in normal tissues, but there was no significant difference (*P*=0.345) (Supplementary Figure S1). These findings were consistent with previous reports (Bandres *et al*, 2009; Oh *et al*, 2011).

## Discussion

Mitchell *et al* (2008) recently reported that miRNAs are detectable in plasma and that circulating miRNAs have the potential to be new biomarkers in patients with prostate cancers. They also demonstrated the high stability of plasma miRNAs after prolonged incubation at room temperature and/or multiple freezing–thawing processes. In addition to this high stability, the characteristics of miRNAs such as tissue-specific miRNA signatures and the availability of many copies per cell would indicate potential advantages as biomarkers (Zen and Zhang, 2010; Kosaka *et al*, 2010a) compared with other nucleic acids, such as circulating DNA and mRNA.

In fact, many studies have demonstrated both diagnostic and prognostic value of plasma/serum miRNAs in various cancers (Mitchell *et al*, 2008; Zen and Zhang, 2010; Kosaka *et al*, 2010a), including GC (Tsujiura *et al*, 2010; Wang *et al*, 2010; Wu *et al*, 2010). Most of these studies assessed plasma/serum levels of miRNAs that were selected on the basis of miRNA profiling data in cancer tissues themselves. Some studies, however, reported that the circulating miRNAs are derived from not only tumour-cell lysis but also active secretion as miRNA-protein complexes (Arroyo *et al*, 2011) and/or cell-derived microvesicle form (Ohshima *et al*, 2010), and consequently the expression patterns in plasma would not be identical to those in cancer cell lines and tumour tissues (Pigati *et al*, 2010; Zen and Zhang, 2010).

Several recent studies have investigated circulating miRNAs as a biomarker for cancers on a miRNA microarray. Zhao *et al* (2010) found a potential miRNA biomarker for breast cancer patients using microarray-based expression comparison between plasma miRNA expression of early stage breast cancer patients and healthy controls. MicroRNAs, however, are involved in many aspects of noncancerous cell biology including physiological modulation and pathological disruption of basic pathways (Ambros, 2004; Bartel, 2004), which strongly suggest the existence of considerable interindividual differences in miRNA expression. To eliminate the effect of individual differences on the results of plasma miRNA microarray analysis, we employed an experimental strategy using pre- and post-operative paired plasma samples of the same cancer patients in this study. In regard to the timing of post-operative blood collections, we have already confirmed that the concentrations of tumour-derived miRNAs were significantly reduced post-operatively and that 1 month seems to be sufficient for clearance of the circulating miRNAs (Tsujiura *et al*, 2010; Komatsu *et al*, 2011), although the kinetics and metabolism of the plasma miRNAs have not yet been clearly elucidated.

In the microarray analysis in this study, we found that concentrations of some miRNAs were markedly decreased in post-operative plasma samples compared with those pre-operatively. After verification of the results by qRT–PCR, two miRNAs (miR-451 and miR-486) were selected for further analyses as potential plasma biomarkers in GC patients, which satisfy the selection criteria of being abundant in plasma and showing a marked decrease in post-operative plasma. In large-scale validation study of the peri-operative paired samples, both plasma miRNAs were found to decrease post-operatively in most cases (90% and 93%, respectively). Meanwhile, in the comparison study between healthy controls and GC patients, the levels of both plasma miRNAs were also found to be significantly higher in GC patients than in healthy controls. The analyses of both plasma miRNAs showed the high AUC values of 0.96 for miR-451 and 0.92 for miR-486 in this study, which would be satisfactory for clinical application.

First, we predicted that miRNAs with high expression in GC tissues would decrease in post-operative plasma samples in the microarray analysis because the majority of circulating miRNAs were considered to be derived from apoptosis and necrosis of cancer cells (Zen and Zhang, 2010). Indeed, some oncogenic miRNAs with high expression in GC tissues, such as miR-25, miR-17 and miR-106a (Yao *et al*, 2009; Tsujiura *et al*, 2010; Wang *et al*, 2010; Wu *et al*, 2010), were markedly decreased in the post-operative plasma samples. However, miR-451 and miR-486 have been reported as tumour-suppressive miRNAs in primary GC tissues (Bandres *et al*, 2009; Oh *et al*, 2011), contrary to our expectations. Bandres *et al* (2009) showed that miR-451 was downregulated in GC tissues, and this alteration was associated with reductions of disease-free and overall survival in GC patients. On the other hand, Oh *et al* (2011) identified miR-486 as a significantly downregulated miRNA in primary GC tumours and GC cell lines, and restoration of miR-486 expression in GC cells caused suppression of several pro-oncogenic traits, including cell proliferation, anchorage-independent growth and cell migration/invasion.

On the other hand, Brenner *et al* (2011) recently described the high-level expression of miR-451 in GC tissue to be predictive of the recurrence of GC. However, the findings reported by Brenner *et al* do not necessarily contradict those of ours, because Brenner *et al* analysed miRNA expression levels of cancer tissues comparing only a good with a poor prognosis group, not comparing those with adjacent normal gastric mucosa.

An additional consideration is the fact that miR-451 expression is abundant in blood cells, because it has been described to be associated with erythroid maturation (Zhan *et al*, 2007). Then, the incorrect collection or treatment of blood samples might result in false high expression of miR-451 in plasma samples. In quantitative RT–PCR analyses, however, only pre-operative samples of GC patients have a tendency to express high miR-451, and also miR-486; therefore, our blood sampling is believed to be adequate.

The reason why the miRNAs with relatively lower expression in GC tissues (Supplementary Figure S1) showed higher plasma concentration than those in healthy controls is unknown at present. There are several possible explanations: the first theory is that some miRNAs might be released selectively from cancer cells to stroma and circulation (Kosaka *et al*, 2010a; Zen and Zhang, 2010). Ohshima *et al* (2010) proposed that the let-7 miRNA family members, which generally act as tumour-suppressive factors and are downregulated in GC tumours, were selectively released into the extracellular environment in highly metastatic GC cell line. A second possible theory is that the plasma miRNAs might also be released from normal tissues by unknown mechanisms (Ribeiro-dos-Santos *et al*, 2010; Zen and Zhang, 2010; Reid *et al*, 2011). Pigati *et al* (2010) suggested that the extracellular miRNA profile is not similar to that of intracellular miRNA, and some of extracellular miRNA might be derived from normal epithelial cells in breast cancer patients. Moreover, in this study, some clinicopathological factors correlated with plasma miRNA level (Supplementary Table S2): the lower concentration of these plasma miRNAs significantly correlated with residual tumour, worse lymphatic invasion, and advanced TNM stage. However, the exact reason for this has not been identified. Kosaka *et al* (2010b) revealed one secretary machinery of miRNAs and their intercellular transfer, and suggested that these circulating miRNAs might have a role as a signalling molecule. Further analysis should clarify the origin of extracellular circulating miRNAs and shed light on the causation of the correlations in the near future.

The key feature of this study is that potential diagnostic biomarkers were discovered by a unique approach comparing pre- and post-operative plasma samples. We believe that this unique approach could provide so-called ‘tailor-made’ tumour markers for individual cancer patients. Personalised tumour markers might realise individualised medicine: confirming the completeness of tumour resection, evaluating the efficacy of cancer therapies, and monitoring disease recurrence.

In conclusion, this study clearly demonstrated that plasma miR-451 and miR-486 could be useful blood-based biomarkers for screening GC patients and monitoring tumour dynamics. These non-invasive blood-based biomarkers could have great potential for use clinically to predict the clinical behaviour of individual cancers and to monitor therapeutic response. Further prospective clinical trials using a variety of plasma miRNAs should be carried out to define the usefulness of the assay for each potential application.

## Figures and Tables

**Table 1:** The miRNAs the level of which were markedly decreased in post-operative plasma

	**MK408**			**MK416**			**MK438**		
	**Before (B)**	**After (A)**	**Ratio (A/B)**	**Before (B)**	**After (A)**	**Ratio (A/B)**	**Before (B)**	**After (A)**	**Ratio (A/B)**
▸hsa-miR-451	55 953.8	853.9	0.01526	65 354.0	5599.2	0.08568	48 529.9	9509.5	0.19595
▸hsa-miR-16	4566.1	119.4	0.02615	10 502.5	1318.3	0.12552	7540.1	964.0	0.12784
▸hsa-miR-486-5p	9843.7	524.4	0.05327	24 219.0	1219.6	0.05036	12 132.8	1798.8	0.14826
▸hsa-miR-25	1029.5	97.1	0.09435	2455.1	272.6	0.11103	1940.8	282.1	0.14533
hsa-miR-19b	2080.8	213.7	0.10271	2987.2	578.5	0.19365	2466.3	533.2	0.21622
▸hsa-miR-17	547.2	84.5	0.15435	1298.6	283.8	0.21856	771.6	87.0	0.11281
▸hsa-miR-106a	553.6	90.3	0.16312	1731.5	266.5	0.15393	852.0	135.0	0.15849
hsa-miR-92b	2071.9	347.9	0.16790	4712.7	664.8	0.14106	3066.2	548.4	0.17886
hsa-miR-92a	5049.3	928.6	0.18390	10 877.6	1475.1	0.13561	6602.9	1188.9	0.18006
hsa-miR-20a	349.6	64.5	0.18448	1036.3	201.9	0.19483	694.7	97.5	0.14033
hsa-let-7d	258.6	87.2	0.33718				427.4	117.1	0.27392
▸hsa-miR-30c	176.7	62.3	0.35255				407.8	130.1	0.31904
▸hsa-let-7a	226.5	89.4	0.39459				298.3	57.9	0.19425
▸hsa-miR-1203	437.8	127.0	0.29007	1171.1	149.2	0.12742	3112.4	206.0	0.06619
hsa-miR-874	412.0	159.9	0.38815	1715.5	88.3	0.05149	1889.5	205.8	0.10892
hsa-miR-484	603.4	254.2	0.42118	1539.4	290.7	0.18881	1090.8	156.0	0.14298
hsa-miR-675	259.4	109.6	0.42248	559.6	127.1	0.22715	1130.2	130.9	0.11582
hsa-miR-1914^*^	259.4	109.6	0.42248	851.2	173.6	0.20395	2114.1	213.6	0.10103
hsa-miR-1228^*^	13 067.0	5671.3	0.43401	27 316.0	3955.6	0.14481	43 298.4	5361.1	0.12382
hsa-miR-3162	186.8	83.0	0.44436	353.0	80.9	0.22933	589.1	102.6	0.17425

Abbreviation: miRNA=microRNA. After (A): post-operative samples.

Before (B): pre-operative samples.

Arrowheads: candidate miRNAs for validation study.
