# Supplementary material for: Detection of gastric cancer-associated microRNAs on microRNA microarray comparing pre- and post-operative plasma
Source: Br J Cancer. 2012 Jan 19;106(4):740–7. doi: 10.1038/bjc.2011.588 (PMC3322946; doi:10.1038/bjc.2011.588)
Supplement: Supplementary Figure S1 [file bjc2011588x1.ppt]

## Slide 1
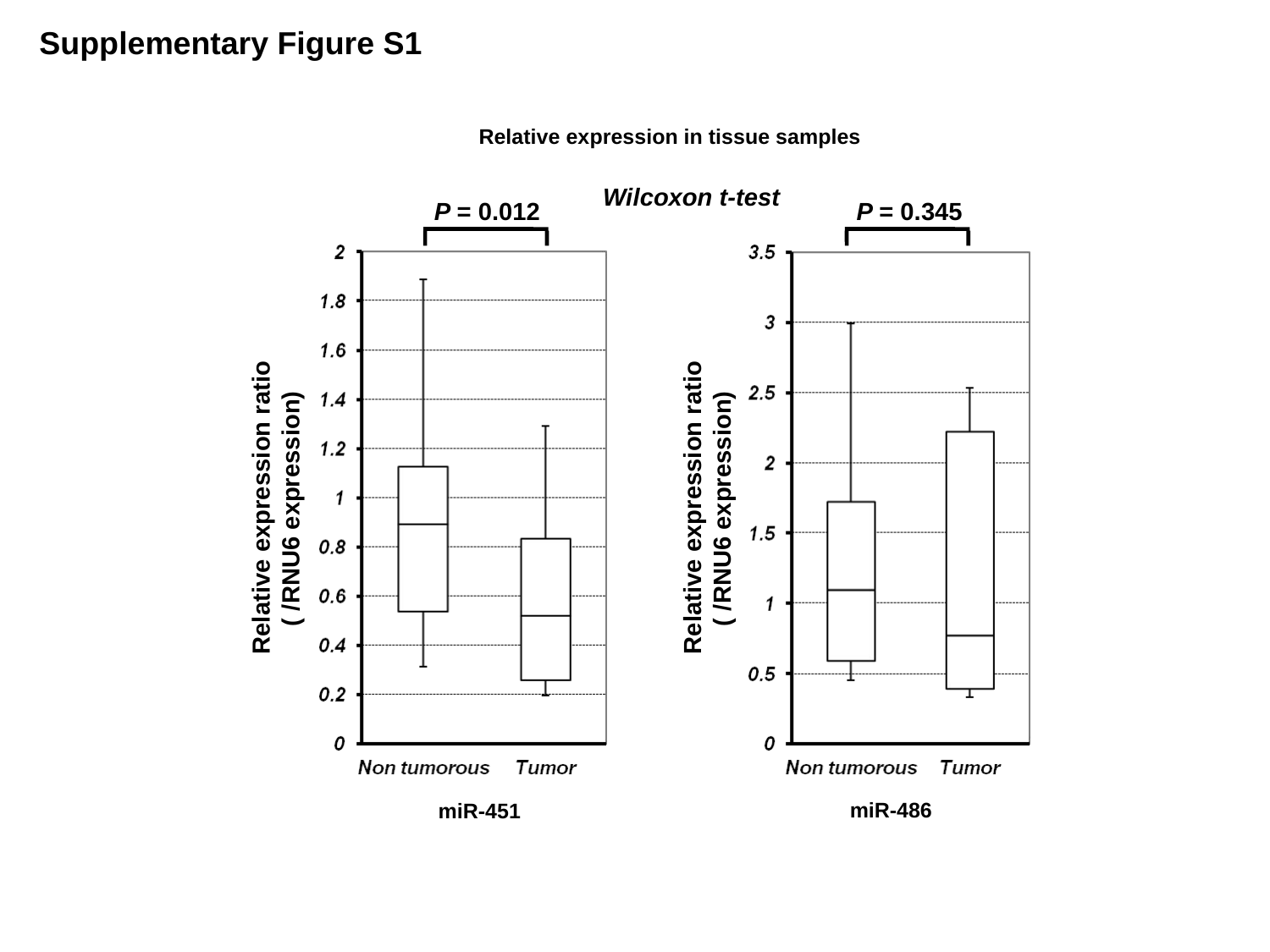

Supplementary Figure S1
Relative expression in tissue samples
Wilcoxon t-test
P = 0.012
P = 0.345
Relative expression ratio
 ( /RNU6 expression)
Relative expression ratio
 ( /RNU6 expression)
miR-486
miR-451
