# Supplementary material for: Detection of gastric cancer-associated microRNAs on microRNA microarray comparing pre- and post-operative plasma
Source: Br J Cancer. 2012 Jan 19;106(4):740–7. doi: 10.1038/bjc.2011.588 (PMC3322946; doi:10.1038/bjc.2011.588)
Supplement: Supplementary Figure Legend [file bjc2011588x2.doc]

**SUPPLEMENTARY FIGURE LEGENDS**

**Supplementary Figure S1**

**Comparison of miR-451 and miR-486 expressions between non-tumorous tissues and GC tissues**

The miRNA expression is presented as the relative expression ratio (the candidate miRNA expression/RNU-6 expression). Both miRNA expressions were found to be relatively lower in GC tissues than in surrounding normal tissues. The upper and lower limits of the boxes and the lines inside the boxes indicate the 75th and 25th percentiles and the median, respectively. The upper and lower horizontal bars denote the 90th and 10th percentiles, respectively.
