# Supplementary material for: Detection of gastric cancer-associated microRNAs on microRNA microarray comparing pre- and post-operative plasma
Source: Br J Cancer. 2012 Jan 19;106(4):740–7. doi: 10.1038/bjc.2011.588 (PMC3322946; doi:10.1038/bjc.2011.588)
Supplement: Supplementary Table S1 [file bjc2011588x3.ppt]

## Slide 1
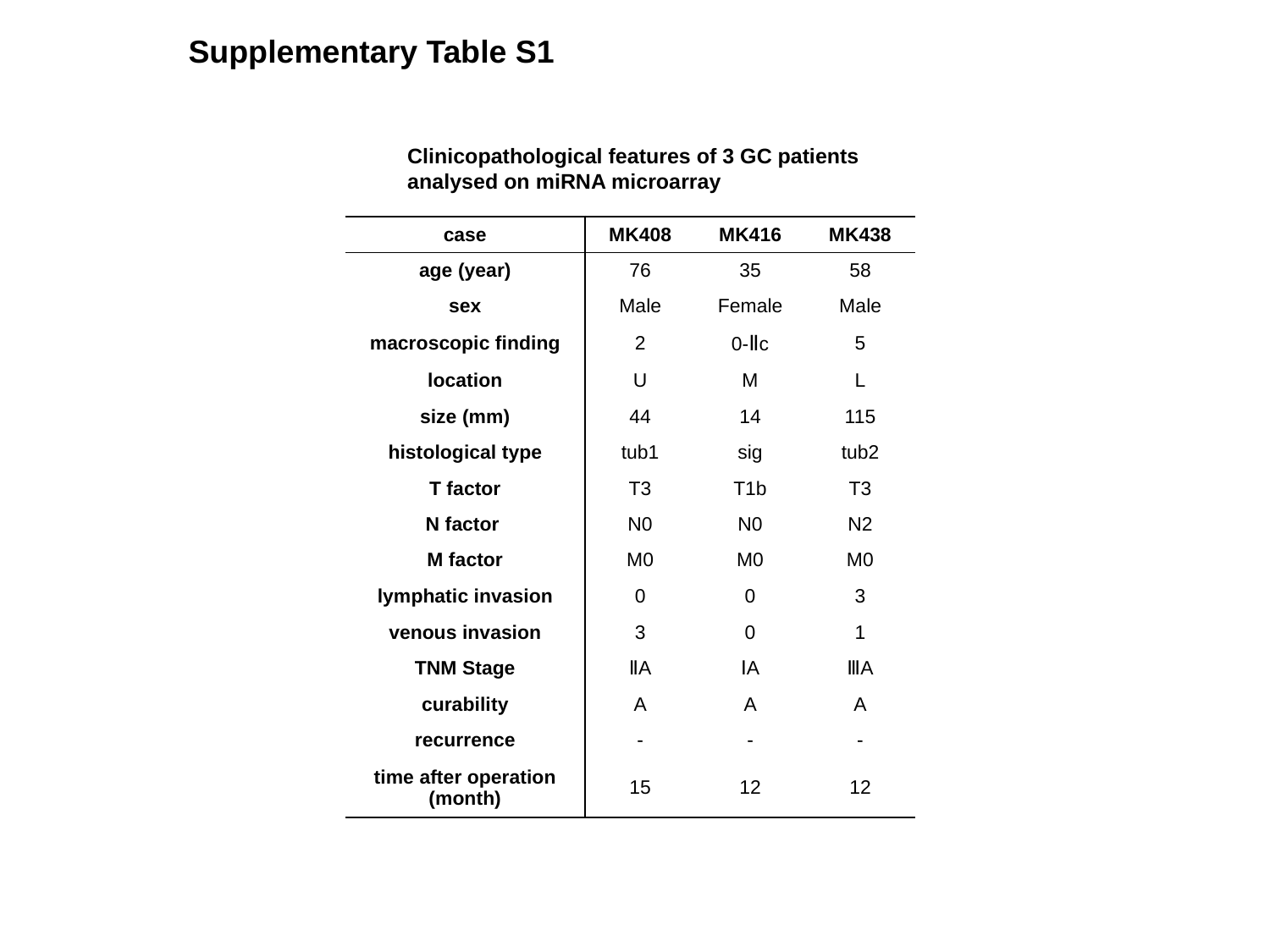

Supplementary Table S1
Clinicopathological features of 3 GC patients
analysed on miRNA microarray
| case | MK408 | MK416 | MK438 |
| --- | --- | --- | --- |
| age (year) | 76 | 35 | 58 |
| sex | Male | Female | Male |
| macroscopic finding | 2 | 0-Ⅱc | 5 |
| location | U | M | L |
| size (mm) | 44 | 14 | 115 |
| histological type | tub1 | sig | tub2 |
| T factor | T3 | T1b | T3 |
| N factor | N0 | N0 | N2 |
| M factor | M0 | M0 | M0 |
| lymphatic invasion | 0 | 0 | 3 |
| venous invasion | 3 | 0 | 1 |
| TNM Stage | ⅡA | ⅠA | ⅢA |
| curability | A | A | A |
| recurrence | - | - | - |
| time after operation (month) | 15 | 12 | 12 |
