# Supplementary material for: Detection of gastric cancer-associated microRNAs on microRNA microarray comparing pre- and post-operative plasma
Source: Br J Cancer. 2012 Jan 19;106(4):740–7. doi: 10.1038/bjc.2011.588 (PMC3322946; doi:10.1038/bjc.2011.588)
Supplement: Supplementary Table S2 [file bjc2011588x4.ppt]

## Slide 1
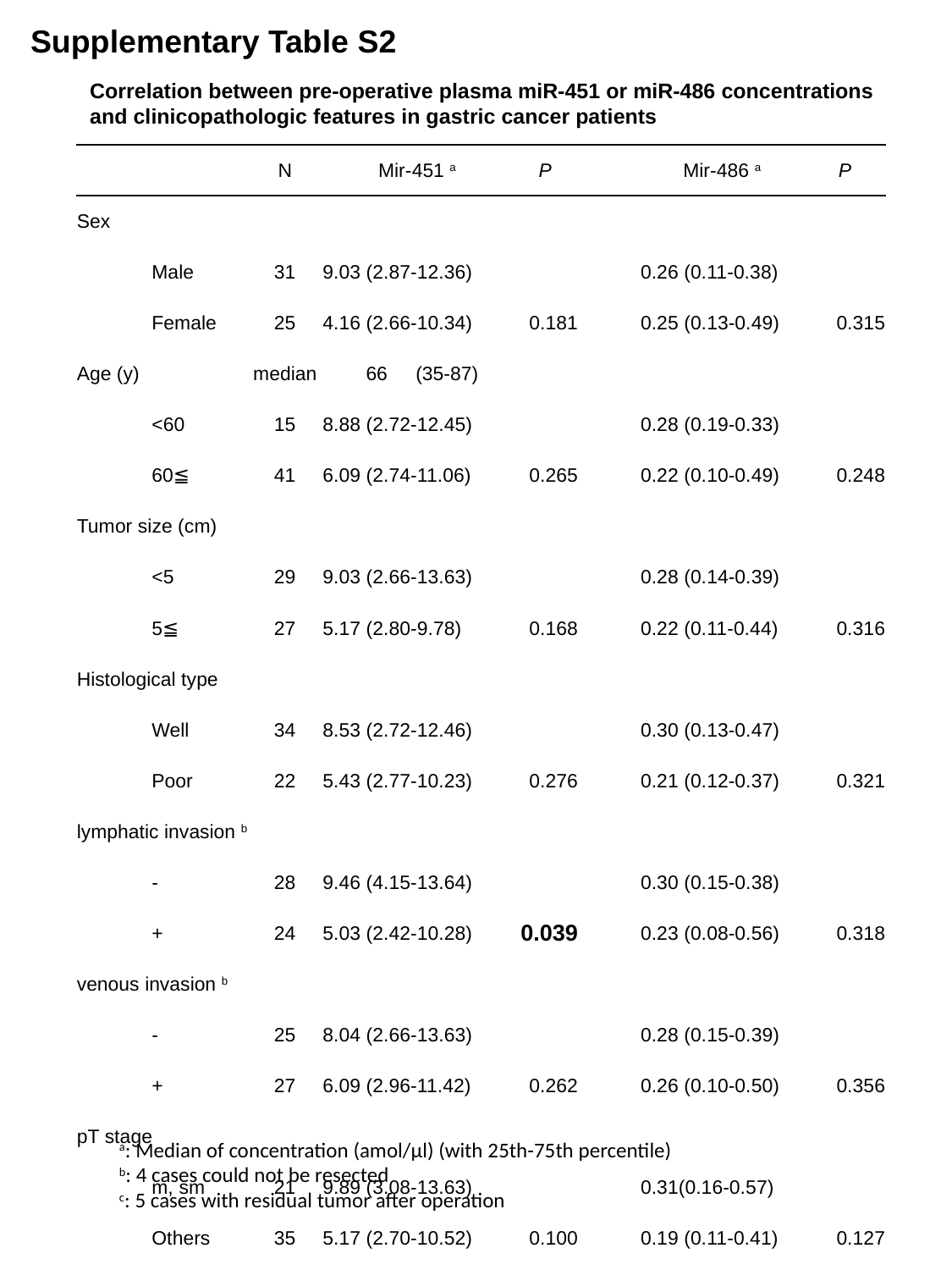

Supplementary Table S2
Correlation between pre-operative plasma miR-451 or miR-486 concentrations
and clinicopathologic features in gastric cancer patients
| | | N | Mir-451 a | P | | Mir-486 a | P |
| --- | --- | --- | --- | --- | --- | --- | --- |
| Sex | | | | | | | |
| | Male | 31 | 9.03 (2.87-12.36) | | | 0.26 (0.11-0.38) | |
| | Female | 25 | 4.16 (2.66-10.34) | 0.181 | | 0.25 (0.13-0.49) | 0.315 |
| Age (y) | | median | 66　(35-87) | | | | |
| | <60 | 15 | 8.88 (2.72-12.45) | | | 0.28 (0.19-0.33) | |
| | 60≦ | 41 | 6.09 (2.74-11.06) | 0.265 | | 0.22 (0.10-0.49) | 0.248 |
| Tumor size (cm) | | | | | | | |
| | <5 | 29 | 9.03 (2.66-13.63) | | | 0.28 (0.14-0.39) | |
| | 5≦ | 27 | 5.17 (2.80-9.78) | 0.168 | | 0.22 (0.11-0.44) | 0.316 |
| Histological type | | | | | | | |
| | Well | 34 | 8.53 (2.72-12.46) | | | 0.30 (0.13-0.47) | |
| | Poor | 22 | 5.43 (2.77-10.23) | 0.276 | | 0.21 (0.12-0.37) | 0.321 |
| lymphatic invasion b | | | | | | | |
| | - | 28 | 9.46 (4.15-13.64) | | | 0.30 (0.15-0.38) | |
| | + | 24 | 5.03 (2.42-10.28) | 0.039 | | 0.23 (0.08-0.56) | 0.318 |
| venous invasion b | | | | | | | |
| | - | 25 | 8.04 (2.66-13.63) | | | 0.28 (0.15-0.39) | |
| | + | 27 | 6.09 (2.96-11.42) | 0.262 | | 0.26 (0.10-0.50) | 0.356 |
| pT stage | | | | | | | |
| | m, sm | 21 | 9.89 (3.08-13.63) | | | 0.31(0.16-0.57) | |
| | Others | 35 | 5.17 (2.70-10.52) | 0.100 | | 0.19 (0.11-0.41) | 0.127 |
| pN stage | | | | | | | |
| | - | 22 | 9.96 (3.80-13.34) | | | 0.30 (0.16-0.57) | |
| | + | 34 | 5.03 (2.66-10.63) | 0.053 | | 0.21 (0.11-0.44) | 0.234 |
| pTMN stage | | | | | | | |
| | Ⅰ+Ⅱ | 33 | 9.03 (4.15-13.63) | | | 0.31 (0.14-0.43) | |
| | Ⅲ+Ⅳ | 23 | 3.86 (2.53-9.29) | 0.016 | | 0.19 (0.08-0.38) | 0.135 |
| recurrence b,c | | | | | | | |
| | - | 35 | 8.04 (3.47-13.05) | | | 0.32 (0.14-0.56) | |
| | + | 12 | 7.02 (2.98-11.33) | 0.341 | | 0.23 (0.12-0.33) | 0.129 |
| residual tumor | | | | | | | |
| | - | 47 | 8.04 (3.04-12.45) | | | 0.31 (0.14-0.46) | |
| | + | 9 | 2.74 (1.70-4.89) | 0.011 | | 0.10 (0.07-0.22) | 0.018 |
a: Median of concentration (amol/μl) (with 25th-75th percentile)
b: 4 cases could not be resected
c: 5 cases with residual tumor after operation
